# Guerbet Alcohols, Ideal Substrates for the Sustainable Production of Branched Esters

**DOI:** 10.3390/ma18225129

**Published:** 2025-11-11

**Authors:** María Claudia Montiel, Fuensanta Máximo, María Gómez, María Dolores Murcia, Salvadora Ortega-Requena, Josefa Bastida

**Affiliations:** Department of Chemical Engineering, Faculty of Chemistry, University of Murcia, Campus of Espinardo, 30100 Murcia, Spain; cmontiel@um.es (M.C.M.); fmaximo@um.es (F.M.); maria.gomez@um.es (M.G.); md.murcia@um.es (M.D.M.); dortega@um.es (S.O.-R.)

**Keywords:** Guerbet alcohol, branched esters, lipase, biocatalysis, emollients, bioreactors

## Abstract

Saturated and branched high molecular weight organic esters are highly valued as emollients in the cosmetic industry due to their superior properties. Their saturated character provides resistance to oxidation and rancidity. Additionally, their branched structure endows them with low melting temperatures, enabling them to remain liquid over a broad temperature range. These esters can be obtained from branched alcohols, branched fatty acids or both, using chemical or enzymatic processes. Among branched alcohols, Guerbet alcohols stand out. Due to their characteristic properties as branched, saturated alcohols with superior oxidative stability and extremely low volatility, they are proposed as excellent substrates for the enzymatic synthesis of these compounds. This study represents the first investigation into the biocatalytic synthesis of three specific esters: those formed between 2-octyl-1-dodecanol (C20 Guerbet alcohol) and the fatty acids myristic (MA), palmitic (PA), and stearic acid (SA). To achieve this, an environmentally sustainable biocatalytic process was developed. The synthesis involves a solvent-free esterification catalyzed by the commercial immobilized lipase, Lipozyme^®^ 435, conducted within a vertically stirred, thermostated batch tank reactor. Optimal conditions for lipase concentration and temperature were established, and the sustainability of the process was successfully quantified using various “green metrics”.

## 1. Introduction

High molecular weight esters (200 to 800 g/mol) represent one of the three most utilized families of emollients in the cosmetics industry, alongside silicone oils and hydrocarbons. Despite silicones exhibiting superior usage characteristics [1,2,3,4], their inherent stability leads to environmental concerns, specifically bioaccumulation and resistance to biodegradation. It has been established that the octamethylcyclotetrasiloxane (D4) is a persistent, bioaccumulative and toxic (PBT) substance, as well as a very persistent and very bioaccumulative (vPvB) substance. Similarly, decamethylcyclopentasiloxane (D5) has also been identified as a vPvB substance [5,6]. As a result, cosmetic regulation has been tightened, leading the European Union to impose a limit of 0.1 wt% on the use of D4 and D5 in rinse-off products, which took effect in January 2020 [7]. Beyond this strict legal regulation, growing consumer environmental awareness suggests that esters will emerge as the most promising emollients, particularly in terms of novel molecular structures and production methodologies.

Among emollients, saturated and branched organic esters of high molecular weight exhibit properties that make them highly valuable for applications in the cosmetic industry. Their saturated character provides resistance to oxidation and rancidity. Furthermore, their branched structure gives them low melting temperatures, which allows them to remain liquid over a wide temperature range [8].

The synthesis of branched esters primarily employs esterification and transesterification. Achieving the desired branched structure requires the specific combination of substrates, which can include both branched and linear compounds like alcohols, diols, polyols, fatty acids, diacids, or linear esters [9,10,11,12,13,14,15,16,17,18,19,20,21,22,23,24,25,26,27,28,29,30,31,32].

Guerbet alcohols possess remarkable properties that make them ideal substrates for the enzymatic synthesis of branched esters in solvent-free media. Because of their branched structure, all esters derived from these alcohols are also branched and, consequently, they are liquid at temperatures below 50 °C up to C32 (up to C24 they are liquid at room temperature) [33,34,35]. This property ensures compatibility with the temperatures required for enzymatic activity in solvent-free processes. Additionally, Guerbet alcohols can be derived from vegetable sources, with high purity and at a reasonable cost.

The SASOL company (Sandton, South Africa, https://www.sasol.com/), for instance, offers Guerbet alcohols with 12 to 32 carbon atoms, which are derived from linear alcohols of vegetable origin. One of these products, commercialized under the Sasol Chemicals registered trademark ISOFOL 20 (INCI: Octyldodecanol) is a medium-spreading hydrolysis-stable emollient, suitable across a wide pH range, and serves as a basic material to produce emollient esters [36]. The main applications of Guerbet alcohols are in the cosmetic and pharmaceutical industries, which gives an idea of the high degree of purity at which they are typically marketed.

Branched esters obtained from Guerbet alcohols and fatty acids are a common component in numerous cosmetic formulations. A variety of suppliers offer these esters, and Table 1 provides a summary of commercial companies and the corresponding trade names available in their catalogs. They are integral components found in a vast number of cosmetic preparations across all categories, utilized by virtually every manufacturer, including those in the high-end sector. Given this widespread use, the objective for both industry and administrative bodies must be the transformation of current production processes into proven sustainable methods for producing these essential consumer goods.

**Figure 1:**
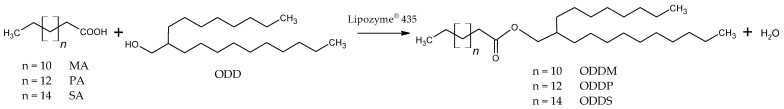
Reaction scheme.

In this context, the main objective of this work is the sustainable synthesis of three commercially important esters of 2-octyl-1-dodecanol (ODD), specifically myristate (ODDM), palmitate (ODDP) and stearate (ODDS), all of which are widely used as ingredients in cosmetic formulations. The proposed methodology involves solvent-free esterification catalyzed by a commercially available immobilized lipase (Figure 1). The use of immobilized biocatalysts is crucial for developing a sustainable process, as it allows the biocatalyst’s reuse over multiple reaction cycles.

## 2. Materials and Methods

### 2.1. Chemicals

Fatty acids, myristic (MA) (≥98%), palmitic (PA) (≥98%) and stearic (SA) (≥95%), were supplied by Merck (Darmstadt, Germany). 2-octil-1-dodecanol (ISOFOL 20) was kindly given by SASOL Chemicals and Lipozyme^®^ 435 lipase, a commercial *Candida antarctica* lipase B (CalB) immobilized on a macroporous acrylic resin, was generously donated by Novozymes Spain S.A. (Madrid, Spain).

### 2.2. Experimental Procedure

The reactions are carried out in a jacketed open-air batch reactor of 50 mL total volume (Vidra FOC 505/2, Barcelona, Spain), stirred at the top with a vertical stirrer (IKA NANOSTAR 75, Staufen, Germany) and connected to a thermostatic bath (Julabo CORIO CD-BC6, Seelbach, Germany).

The reactor contains a mixture of the two substrates in stoichiometric proportions, with a total amount of 20 g, and no solvent is utilized. The temperature of the system varied between 70 and 90 °C, within the range where the substrate mixture is liquid and exhibits perfect homogeneity. Biocatalyst concentrations ranging from 1.25 to 5% (*w*/*w*), measured in percentage of the total substrate mass, were tested. It is also noteworthy that the stirrer speed was set at 350 rpm throughout all synthesis assays.

In order to monitor the progression of the reaction, the acid value of multiple samples extracted from the reactor during the esterification process was determined. The acid value (AV) is defined as the milligrams of potassium hydroxide required to neutralize the free acids present in 1 g of sample [37]. Consequently, conversion can be calculated as follows:

$$Conversion\left(\%\right)=\frac{{AV}_{0}-{AV}_{t}}{{AV}_{0}}\times 100$$

where AV_0_ is the acid value determined at the beginning of the reaction and AV_t_ the acid value of a sample taken at a certain time.

### 2.3. Recovery and Reuse of the Biocatalysts

After reaching the reaction time required to achieve maximum conversion, the agitation process was halted. Thereafter, the immobilized enzyme was permitted to settle, and the products were extracted from the reactor utilizing a pipette. The subsequent reaction cycle is initiated by the addition of fresh substrates to the reactor, without undergoing treatment or the washing of the enzyme. Storage at 4 °C is required if the recovered biocatalyst is not used immediately.

### 2.4. Density and Viscosity Determination

Density and viscosity of all the prepared esters were measured with a DMA 5000 M density meter/LOVIS 2000 M rolling-ball viscometer from Anton Paar (Graz, Austria). The measurements were conducted at three temperatures, 70, 80 and 90 °C.

## 3. Results and Discussion

### 3.1. Influence of Enzyme Concentration

In biocatalytic reactions, it is of vital importance to optimize the amount of enzyme to be used, as its high price is often a decisive factor in the development of an economically viable process. Normally, an increase in the amount of biocatalyst improves the reaction rate, but it can also have negative effects on the synthesis by increasing diffusional limitations and preventing adequate stirring of the reaction mixture.

Therefore, it is necessary to study the influence that increasing amounts of enzyme have on the evolution of the reaction, to determine the amount that provides an adequate rate and avoids the negative effects associated with an excess of solid in the reaction mixture.

**Figure 2:**
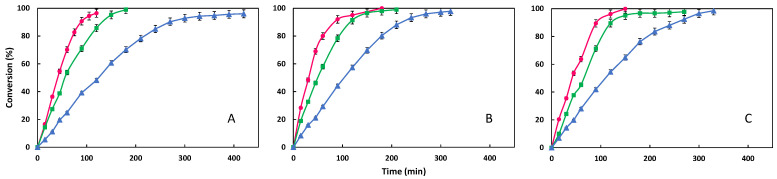
Evolution of conversion with time for synthesis of ODDM (**A**), ODDP (**B**) and ODDS (**C**) at different lipase concentration: (▲) 1.25%, (■) 2.5%, (●) 5% (70 °C, 20 g of substrates, 350 rpm; molar ratio 1:1).

In this study, the synthesis of three esters derived from palmitic, myristic and stearic acids with C20 Guerbet alcohol, 2-octyl-1-dodecanol, has been developed. In all of them, three amounts of Lipozyme^®^ 435 lipase (0.25, 0.5 and 1 g) have been tested, corresponding to concentrations of 1.25, 2.5 and 5% (*w*/*w*) with respect to the total amount of initial substrates. The results obtained are shown in Figure 2, which illustrates the evolution of the conversion with time for the various tests conducted with increasing amounts of enzyme for each ester obtained. The comparison between them will be made by analyzing the average reaction rate in the first 30 min, as well as the time required to reach a conversion of more than 95% (Table 2).

In relation to the average rate in the initial thirty minutes, it can be observed that, for the three esters under study, an increase in the amount of lipase from 0.25 to 0.5 g has a greater effect on this parameter than an increase from 0.5 to 1 g, with this effect being more pronounced for ODDM. Conversely, the time required to achieve conversions exceeding 95% is significantly reduced for changes from 0.25 to 0.5 g lipase (reductions of 240, 120 and 150 min for ODDM, ODDP and ODDS, respectively), and to a lesser extent when increasing from 0.5 to 1 g (reduction of 30 min).

In view of these results, it can be concluded that an increase in the quantity of Lipozyme^®^ 435 from 0.25 to 0.5 g is recommended, since the improvement in the two parameters analyzed is significant; however, the doubling of the quantity of lipase from 0.5 to 1 g does not improve the results in the same proportion, especially with regard to the reduction in the time required to achieve conversions of over 95%. Furthermore, given the high cost of lipase, the increased cost of working with 1 g instead of 0.5 g would be prohibitive considering the reduced improvement in results. Therefore, 0.5 g of Lipozyme^®^ 435, corresponding to a concentration of 2.5% (*w*/*w*) of the total amount of substrates, is selected as the optimal amount.

The findings of this study are consistent with the extant literature, which demonstrates that most researchers operate with lipase concentrations of 2.5% (*w*/*w*) or lower [9,16,20,26,27,29]. Only a small number of studies reports using concentrations slightly exceeding this value with some employing a marginal excess of this concentration [11,22,24,25,30]

### 3.2. Influence of Temperature

Temperature affects the biocatalytic reactions by balancing two factors: reaction enhancement and enzyme stability. Higher temperatures increase the reaction rate and substrate solubility, whilst simultaneously reduce medium viscosity and promote diffusion of substrates. It mitigates mass transport limitations and improves the contact between the substrates and the biocatalyst [14]. Conversely, elevated temperatures can cause enzyme inactivation by damaging its three-dimensional structure, leading to a substantial decline in operational stability and reaction rate [38]. Therefore, enzymes exhibit an optimal temperature range where their efficiency is maximized. For the free CalB lipase, this range is typically between 50 and 60 °C [39]. However, it is widely reported that immobilizing this lipase on a suitable support can dramatically increase its thermal stability, extending the optimal range up to 90 °C [25].

Consequently, it is imperative to analyze the influence of temperature on the progression of the reaction in order to select a temperature that ensures an adequate reaction rate, thereby reducing diffusional limitations without compromising lipase stability.

**Figure 3:**
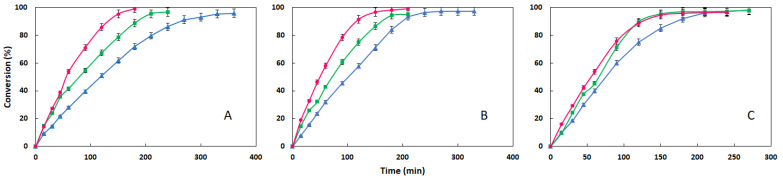
Evolution of conversion with time for synthesis of ODDM (**A**), ODDP (**B**) and ODDS (**C**) at different temperatures: (▲) 70 °C, (■) 80 °C, (●) 90 °C (2.5% (*w*/*w*) of lipase, 20 g of substrates, 350 rpm; molar ratio 1:1).

For this study, synthesis experiments were conducted at three different temperatures (70, 80 and 90 °C), maintaining the optimum lipase concentration determined previously (2.5% (*w*/*w*)). The methodology followed the procedure detailed in Section 2.2, and the results are summarized in Figure 3.

The average reaction rate in the first 30 min, as well as the time required to reach a conversion of more than 95% are the parameters used to compare the results (Table 3).

A close analysis of the results indicates that increasing the temperature enhances the average reaction rate during the initial thirty minutes, while concomitantly reducing the time required to achieve conversions above 95%. The observed increase in the average rate does not appear to be proportional to the temperature rise, as it is more pronounced from 70 to 80 °C than from 80 to 90 °C for ODDM and ODDP, while for ODDS it remains similar for both intervals. The influence on required time for conversions above 95% is also different for the three esters. In the synthesis of ODDM and ODDP, there is a progressive reduction in time with increasing temperatures, while in the synthesis of ODDS there is no drawdown with rising temperature from 80 to 90 °C.

In principle, an economic study could be considered necessary to elucidate whether the higher heating requirement associated with synthesis at 90 °C results in an energy cost that is not compensated by the reduction in reaction time. However, previous research [31] has demonstrated that the cost of the lipase has the most significant impact on the process, followed by the cost of the substrates, and that the heating energy costs are comparable for temperatures of 80 and 90 °C.

Therefore, based on the analysis of the obtained results, 90 °C can be selected as the optimum temperature for the synthesis of ODDM and ODDP and 80 °C for the synthesis of ODDS.

### 3.3. Comparison of the Three Esters at Different Temperature: Influence of Acid Moiety

**Figure 4:**
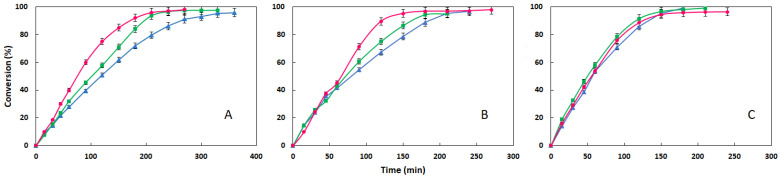
Evolution of conversion with time for synthesis of ODDM (▲), ODDP (■) and ODDS (●) at different temperatures: (**A**) 70 °C, (**B**) 80 °C, (**C**) 90 °C (2.5% (*w*/*w*) of lipase, 20 g of substrates, 350 rpm; molar ratio 1:1).

Since the esters synthesized in this work share the same alcohol (2-octyl-1-dodecanol) but employ fatty acids with increasing chain lengths, the influence of the acid chain length on the reaction system’s behavior is a key aspect for analysis. The acid chain length impacts the reaction progress in two primary ways: longer acid moieties generate a more hydrophobic environment in the system that can affect lipase activity while also increase the viscosity of the reaction medium, which hinders the diffusion of reactants and products. The results demonstrating this influence are presented in Figure 4, grouped according to the reaction temperature.

In the experiments carried out at 70 °C, the best results in terms of both initial speed and reaction time correspond to the synthesis of ODDS, with conversions of over 95% achieved after 210 min. For esters derived from palmitic and myristic acid, the values obtained after the same period are 93.54% and 79.62%, respectively. These results demonstrate that the predominant factor influencing the development of the reaction is the hydrophobicity of the medium, with greater lipase activity observed as the acid chain lengthens. The hydrophobicity of the medium can be quantified based on the octanol–water partition coefficient of the species involved: 8.23 for stearic acid, 7.17 for palmitic acid, and 6.1 for myristic acid. These values correspond to increasing hydrophobicity as the chain length increases.

As the temperature increases, the differences in reaction progress among the three esters diminish. For experiments carried out at 90 °C, the three curves progress similarly, achieving over 95% conversion for the three esters after 210 min. These results demonstrate that as the temperature rises, the influence of hydrophobicity diminishes and that of viscosity increases, resulting in a decrease in diffusional limitations, which improve reaction evolution. This is supported by the data in Table 4, which confirms that increasing the temperature from 70 to 90 °C reduces the measured viscosity by approximately 34%.

### 3.4. Study of Enzyme Reuse

The economic profitability of catalytic processes is often significantly compromised by the cost of the enzyme. To overcome this, immobilized enzymes are used to facilitate the separation and subsequent reuse of the biocatalyst in successive reaction cycles. However, this strategy is only effective if the enzyme is thermally and operationally stable enough to maintain its catalytic activity after each use.

**Figure 5:**
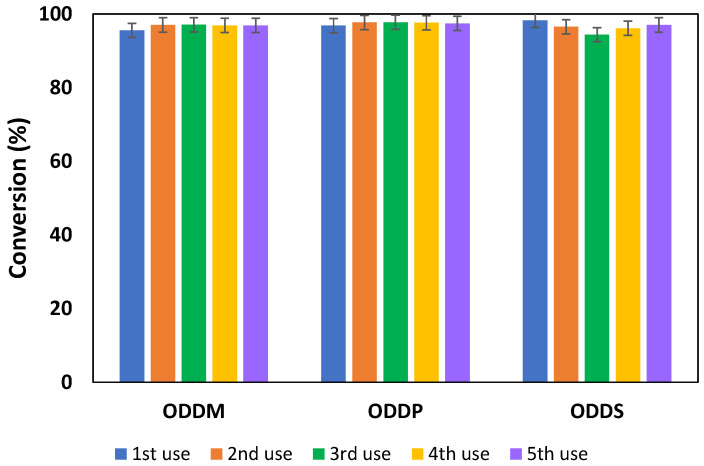
Reusability of the biocatalysts Lipozyme^®^ 435 for the synthesis of ODDM, ODDP and ODDS under the best operational conditions: 2.5% *w*/*w*, 20 g of substrates, 350 rpm; molar ratio 1:1, 90 °C (ODDM and ODDP), 80 °C (ODDS).

In order to assess this operational stability, five successive synthesis tests were performed on each of the esters under study, following the procedure outlined in Section 2.2. After each cycle, the lipase was recovered from the reaction medium, as detailed in Section 2.3, and used for the subsequent assay. The results of this reusability study are shown in Figure 5.

As demonstrated in Figure 5, the lipase exhibits excellent operational stability, retaining its catalytic activity after five reaction cycles with a similar conversion of over 95% achieved at 240 min in all experiments. From an economic point of view, this success immediately enables the reduction in the lipase cost by a factor of five, significantly boosting the process’s profitability. In view of these positive results, it is reasonable to expect the lipase to maintain its activity over a greater number of cycles, a sustained performance already documented in similar systems [40].

### 3.5. Study of Environmental Sustainability

The concept of sustainability has evolved from an abstract concept to a quantifiable entity, as evidenced by the utilization of “green metrics” to measure its impact. Since the seminal work of Sheldon in 1992 on the E-factor, a range of indexes have been developed. This expansion of metrics directly reflects the increasing concern for environmental protection and the growing commitment to developing environmentally sustainable processes [41,42,43,44]. By calculating these metrics, the process sustainability can be comprehensively evaluated based on three fundamental aspects: efficiency in the use of resources (primarily materials and energy); environmental, health and safety (EHS) impacts; and life cycle assessment (LCA) considerations.

A substantial number of “green metrics” are delineated in the extant literature [42,45,46,47,48]. Among these, atom economy (AE), E-factor (EF), complete E-factor (cEF), carbon mass efficiency (CME), and process mass intensity (PMI) are considered particularly suitable for measuring the sustainability of biocatalytic processes [26,27,29,30,49]. Table 5 illustrates the range of calculated sustainability along with their respective values.

Atom Economy is a quantitative metric that indicates the proportion of the raw materials used that are incorporated into the final product, without being lost in the formation of other unwanted products. This is a theoretical number, calculated based on the use of stoichiometric quantities of reagents and 100% yields in the reactions. The value obtained for the different syntheses in this study is slightly below 100% due to the formation of water, indicating both a high utilization of substrates and a highly sustainable process.

The E-factor is a metric used to quantify the amount of waste generated in the process, calculated per kilogram of product produced. It is notable that the solvent used is not taken into consideration, including water. The findings of this study demonstrate that the processes under investigation are highly sustainable, generating only a negligible amount of waste, which corresponds to the lipase that has been discarded after five cycles of use.

The complete E-factor also quantifies the amount of waste generated, with the key caveat that it must consider all solvents used, including water. The cEF values obtained for the three syntheses studied are very low and similar to the E-factor values. This is expected since the reactions are carried out in solvent-free systems, meaning the only liquid accounted for in the cEF calculation is the water generated as a product of esterification.

As deduced from the formula presented in Table 5, Carbon Mass Efficiency is defined as the percentage of carbon from the reactants that remains into the final product. An ideal reaction system is one with a CME equal to 100%; a lower value indicates that a proportion of the carbon from the substrates is lost to the waste stream or emissions. This metric is calculated based on the stoichiometry and the carbon content from the reactants that is incorporated into the final product. As with Atom Economy, the CME does not account for waste generated during the process.

The Process Mass Intensity (PMI) was first established in 2006 by the Environmental Protection Agency (EPA) and the American Chemical Society’s Green Chemistry Institute (GCI) [41]. This indicator is a comprehensive assessment tool that evaluates process efficiency by considering all materials and water utilized, including workup chemicals. However, the PMI does not address energy consumption, safety, or environmental impact. The values obtained in this research are close to one, which indicates the high sustainability of the process.

## 4. Conclusions

This comprehensive study successfully details the sustainable synthesis of three key Guerbet alcohol esters2-octyl-1-dodecanoyl myristate (ODDM), palmitate (ODDP), and stearate (ODDS)—utilizing the immobilized lipase Lipozyme^®^ 435 within a highly desirable solvent-free reaction system. This methodology provides a demonstrable pathway toward more environmentally benign production, particularly within the cosmetic sector where these materials are extensively used.

For these three esters, the optimum catalyst concentration was determined to be 2.5%, with an optimum temperature of 90 °C for ODDM and ODDP, and 80 °C for ODDS. The variation in the length of the acid residue has been shown to affect the system differently, depending on the temperature. At low temperatures, the medium’s hydrophobicity predominates, a property that increases in proportion to the length of the acid chain, thereby enhancing lipase activity. Conversely, at elevated temperatures, the reduction in viscosity assumes greater significance, as it mitigates limitations imposed by diffusion.

The immobilized lipase demonstrated outstanding stability, maintaining a consistently high conversion rate (over 95%) across five successive reaction cycles for all esters. This sustained performance has a significant economic impact enhancing process profitability and commercial feasibility.

The sustainability of the proposed syntheses was quantitatively confirmed through the calculation of established green metrics (AE, EF, cEF, CME, PMI). The values obtained for these indicators demonstrate the high environmental sustainability of the proposed syntheses, both in relation to the waste generated and the use of raw materials.

## Figures and Tables

**Table 1:** Summary of branched esters obtained from Guerbet alcohols and fatty acids, commercial suppliers and trade name.

Trade Name	Supplier
INCI name: octyldodecyl myristate	
PARYOL MIRISTIL 8–12	A&A Fratelli Parodi, Genoa, Italy
Bernel Ester 2014	Alzo International, Freehold, NJ, USA
Dermol 2014	
Wickenol 142	
Octyldodecyl Myristate	Blue Sun International, Miami, FL, USA
Corum 5024	CORUM, La Chaux-de-Fonds, Switzerland
ERCAREL ODM V	ErcaWilmar, São Paulo, Brazil
HEST ODM	Ethox Chemicals, Greenville, SC, USA
MOD MB	Gattefossé, Saint-Priest, France
ODM 100KC	KCI, San Antonio, TX, USA
ODM	Kokyu Alcohol Kogyo, Nagoya, Japan
NIKKOL ODM-100	NIKKOL GROUP (Nikko Chemicals), Tokyo, Japan
Natura-tec Ultrafeel ODM	Natura-Tec, Fremont, CA, USA
Paester^TM^ ODM	Patech Fine Chemicals, Elmsford, NY, USA
RITAMOLLIENT ODDM	RITA Corporation, Crystal Lake, IL, USA
Saboderm ODM	Sabo, Levata, Italy
DUB MOD	Stearinerie Dubois, Boulogne-Billancourt, France
INCI name: octyldodecyl palmitate	
Corum 5025	CORUM, La Chaux-de-Fonds, Switzerland
INCI name: octyldodecyl stearate	
Corum 5026	CORUM, La Chaux-de-Fonds, Switzerland
HEST ODS	Ethox Chemicals, Greenville, SC, USA
Ceraphyl^TM^ ODS ester	Ashland, Wilmington, DE, USA

**Table 2:** Comparison of results obtained for the different amount of lipase tested.

Ester	Amount of Lipase (g)	Average Rate at 30 min (min^−1^)	Time to Conversion up 95% (min)
ODDM	0.25	0.373	390
	0.5	0.914	150
	1	1.213	120
ODDP	0.25	0.533	270
	0.5	1.091	150
	1	1.614	120
ODDS	0.25	0.476	300
	0.5	0.808	150
	1	1.185	120

**Table 3:** Comparison of results obtained for the different temperatures tested.

Ester	Temperature (°C)	Average Rate at 30 min (min^−1^)	Time to Conversion up 95% (min)
ODDM	70	0.490	360
	80	0.794	210
	90	0.914	150
ODDP	70	0.521	240
	80	0.857	210
	90	1.091	150
ODDS	70	0.617	210
	80	0.808	180
	90	0.977	180

**Table 4:** Values of viscosity measured for the three esters studied.

Ester	Temperature (°C)	Dynamic Viscosity (cp)	Cinematic Viscosity (mm^2^/s)
ODDM	70	5.975	7.269
	80	4.794	5.879
	90	3.929	4.857
ODDP	70	6.880	8.365
	80	5.492	6.730
	90	4.479	5.533
ODDS	70	8.492	10.330
	80	6.851	8.402
	90	5.576	6.893

**Table 5:** Green metrics determined to analyze the sustainability of the biocatalytic synthesis of ODDM, ODDP and ODDS after five uses of the enzymes.

Green Metric	Formula	ODDM	ODDP	ODDS
Atom Economy (AE) (%)	$AE=\frac{molecular\;weight\;of\;desired\;product}{\sum molecular\;weight\;of\;all\;reactants}\times 100$	96.57	96.76	97.00
E-Factor (EF)	$EF=\frac{kg\;of\;waste}{kg\;of\;desired\;product}$	0.041	0.032	0.010
Complete E-factor (cEF)	$cEF=\frac{kg\;of\;waste\;(including\;water)}{kg\;of\;desired\;product}$	0.076	0.067	0.041
Carbon Mass Efficiency (CME) (%)	$CME=\frac{kg\;of\;carbonated\;desired\;product}{kg\;of\;carbonated\;reactants}\times 100$	96.70	97.55	99.58
Process Mass Intensity (PMI)	$PMI=\frac{kg\;of\;all\;materials}{kg\;of\;desired\;product}$	1.076	1.071	1.005

## Data Availability

The raw data supporting the conclusions of this article will be made available by the authors on request.

## Abbreviations

The following abbreviations are used in this manuscript:

AE	Atom economy
AV	Acid Value
CalB	*Candida antarctica* lipase B
cEF	Complete E-factor
CME	Carbon mass efficiency
D4	Octamethylcyclotetrasiloxane
D5	Decamethylcyclopentasiloxane
EF	E-factor
EHS	Environmental, health and safety
EPA	Environmental Protection Agency
GCI	Green Chemistry Institute
LCA	Life cycle assessment
MA	Myristic acid
ODD	2-octyl-1-dodecanol
ODDM	2-octyl-1-dodecanoyl myristate
ODDP	2-octyl-1-dodecanoyl palmitate
ODDS	2-octyl-1-dodecanoyl stearate
PA	Palmitic acid
PBT	Persistent, bioaccumulative and toxic
PMI	Process mass intensity
SA	Stearic acid
vPvB	Very persistent and very bioaccumulative
